# Laparoscopic right hemicolectomy in situs inversus totalis with cecal carcinoma: a case report and literature review

**DOI:** 10.3389/fonc.2025.1668016

**Published:** 2025-11-13

**Authors:** Bowen Su, Yang Zhong, Jiahan Chen, Hongpeng Tian, Tianhao Wu, Guangjun Zhang

**Affiliations:** Second Department of Gastrointestinal Surgery, Hepatobiliary, Pancreatic and Intestinal Diseases Research Institute,The Affiliated Hospital of North Sichuan Medical College, National Clinical Key Specialty (General Surgery), Sub-center of National Clinical Research Center for Digestive Diseases, Sichuan Clinical Research Center for Digestive Diseases, Nanchong, Sichuan, China

**Keywords:** situs inversus totalis, colorectal cancer, laparoscopic radical resection, case report, tomography angiography

## Abstract

**Background:**

Our patient exhibited situs inversus totalis (SIT)—a developmental quirk resulting in right–left transposition of all visceral organs, including the heart, liver, and spleen. While prior case reports have described colorectal carcinoma in individuals with SIT, to our knowledge, this case represents an exceptionally rare presentation of primary cecal carcinoma within this anatomical context. This case describes a patient undergoing laparoscopic right hemicolectomy for adenocarcinoma of the cecum.

**Case presentation:**

A patient presented to our institution in May 2024. Subsequent diagnostic workup confirmed a diagnosis of cecal carcinoma. Given the absolute contraindication for bowel preparation secondary to complete bowel obstruction, along with radiologically confirmed cecal malignancy and elevated serum tumor markers, a multidisciplinary consensus was reached to proceed with laparoscopic right hemicolectomy after obtaining proper informed consent from the patient’s family. Pathology confirmed a T3N0M0 well-differentiated adenocarcinoma. Twelve-month consecutive postoperative follow-up data confirmed the absence of surgical complications such as anastomotic leakage or infection, as well as no clinical or radiographic evidence of disease recurrence.

**Conclusion:**

Although the reversed anatomy in SIT patients presents inherent technical challenges for laparoscopic surgery, this minimally invasive approach can still achieve comparable safety and efficacy to conventional procedures when performed by experienced surgeons with adequate anatomical understanding.

## Introduction

Situs inversus totalis (SIT) is an uncommon congenital condition, observed in approximately 1 out of every 5,000 to 10,000 live births, reflecting an uncommon anatomical anomaly (1). Due to the frequent association between internal visceral transposition and congenital anatomical anomalies, surgical intervention may present significant technical difficulties, particularly in laparoscopic approaches, where the altered anatomy may compromise spatial orientation and increase the risk of iatrogenic injuries. The coexistence of colorectal cancer and SIT is globally rare, with cecal carcinoma in this anatomical variant being extraordinarily scarce.

## Case presentation

**Figure 1:**
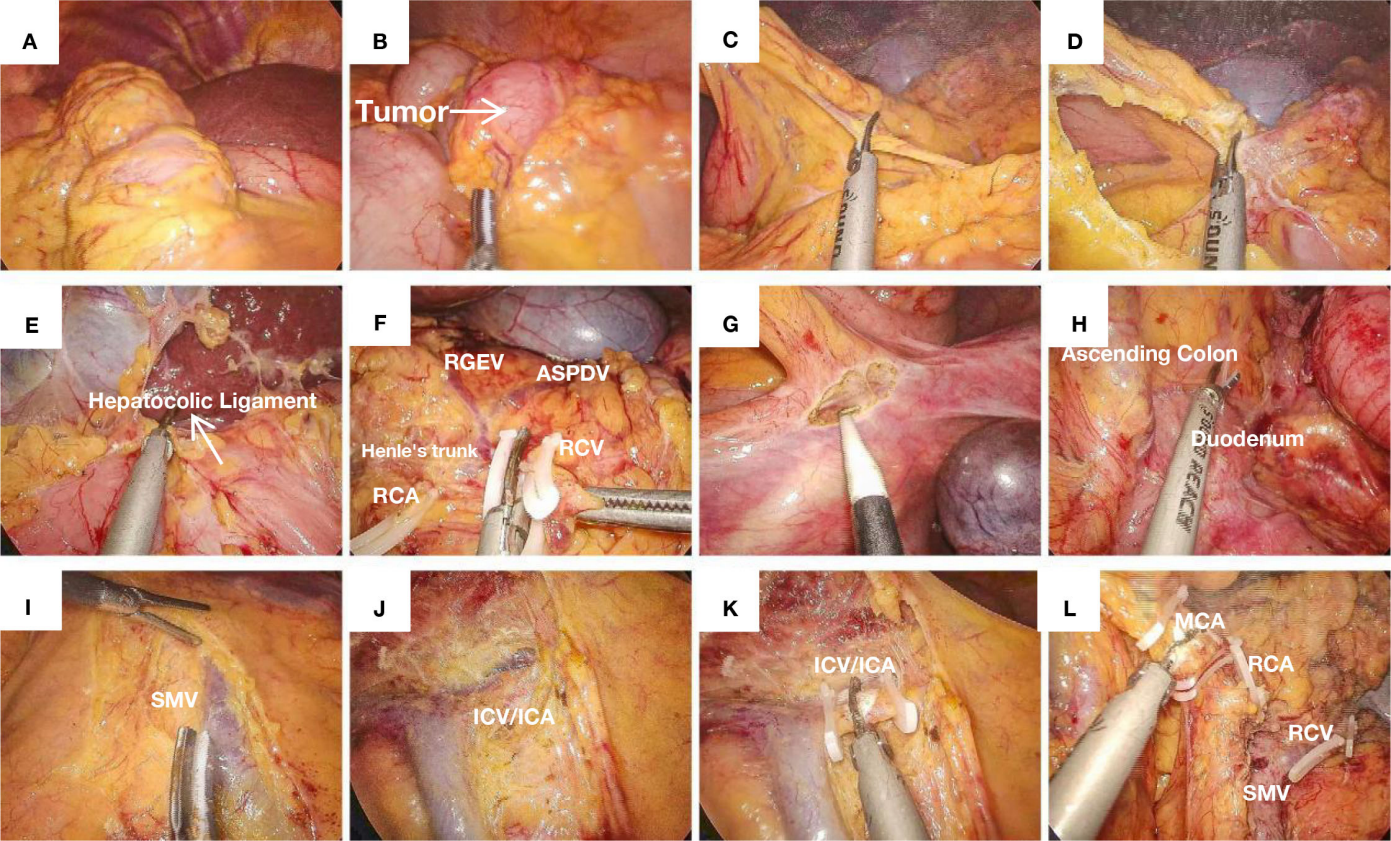
**(A)** SIT: The liver is located on the left side of the abdominal cavity. **(B)** A tumor located in the cecum causing obstruction, with proximal bowel dilation. **(C)** Opening the greater omentum. **(D)** Opening the lesser omentum along the plane parallel to the gastroepiploic vessels. **(E)** Transection of the hepatocolic ligament. **(F)** The classic Henle’s trunk, with preservation of the RGEV and the ASPDV, while transecting the RCV. **(G)** Incision and dissection along the avascular plane above the cecum, separating the gonadal vessels and ureter. **(H)** Exposure of the duodenum and ascending colon. **(I)** Skeletonization of the posterior sheath of the SMV. **(J)** Exposure of the SMV. **(K)** Ligation and transection of the ileocolic vessels. **(L)** Ligation and transection of the middle colic vessels.

A 76-year-old woman presented to our institution in May 2024 due to persistent abdominal distension accompanied by left-sided abdominal pain for over 10 days. As shown in **Figures 1A, B**, abdominal computed tomography (CT) revealed SIT with cecal carcinoma complicated by intestinal obstruction and metastatic lymphadenopathy. Laboratory tests revealed elevated tumor markers [carcinoembryonic antigen (CEA) 17.2 ng/mL, CA-199–58 U/mL] and decreased hemoglobin (10.6 g/dL), with otherwise normal findings. After obtaining informed consent from the family and multidisciplinary team (MDT) evaluation, the patient underwent a laparoscopic right hemicolectomy with a standard technique. To our knowledge, this case represents the second documented instance of cecal carcinoma in a patient with SIT reported in the literature since the inaugural case was described by Hirano et al. in 2015 (2).

**Figure 2:**
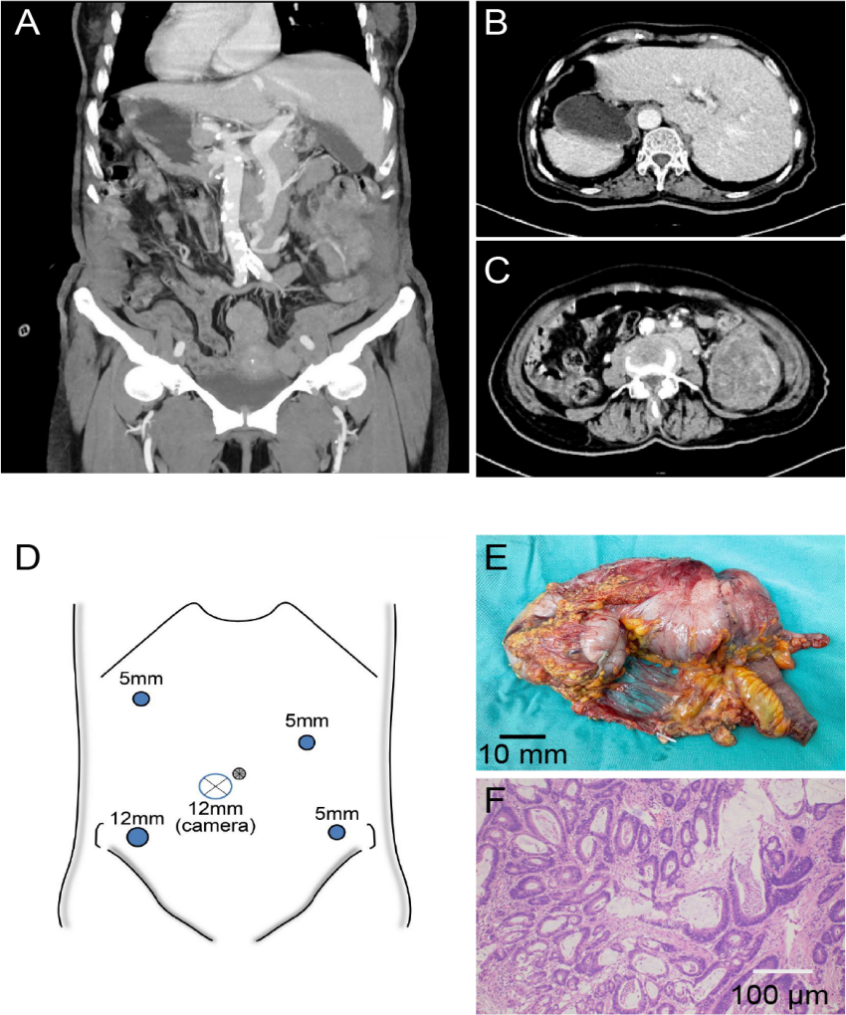
**(A)** Three-dimensional reconstructed coronal view of the abdomen demonstrating situs inversus totalis (SIT), with the liver located on the patient’s left side and the spleen on the right. **(B)** Axial CT reveals SIT, with the liver located on the patient’s left side and a tumor in the cecum also situated on the patient’s left side. **(C)** Axial CT showing SIT with cecal mass and obstruction. **(D)** Layout of the surgical trocar placement. **(E)** Postoperative view of the tumor specimen. **(F)** Pathological image of the tumor.

General endotracheal anesthesia was first administered, after which the patient was positioned in supine lithotomy configuration for the procedure, and a five-port approach was established for laparoscopic access as presented in **Figure 2D**. The trocar configuration was strategically modified to accommodate the mirrored anatomy, with the assistant’s ports positioned on the mirrored side to prevent right-hand instrument crossing between the surgeon and the assistant: the observation port is located below the umbilicus and 2cm to the patient’s right; the primary operating ports are positioned at McBurney’s point (12mm trocar) and two fingerbreadths below the costal margin along the left midclavicular line (5mm trocar); the auxiliary operating ports are situated 1cm above the anti-McBurney’s point (5mm trocar) and four fingerbreadths below the costal margin along the right midclavicular line (5mm trocar).

**Figures 1A, B**, shows the liver is located on the left side of the abdominal cavity and a tumor located in the cecum causing obstruction, with proximal bowel dilation. The operation commenced with cephalad dissection, where meticulous separation of the greater omentum was performed parallel to the gastroepiploic vascular arcade, thereby gaining entry into the lesser peritoneal sac (**Figures 1C, D**). **Figure 1E** shows transection of the hepatocolic ligament. Dissection extended rightward to the splenic flexure and leftward to the infra-pyloric region. During the surgery, it was found that the superior mesenteric vein (SMV) exhibited an anomalous course to the left of the superior mesenteric artery (SMA), presenting as a mirror-image anatomical variant from the surgeon’s perspective. The right gastroepiploic vessels (RGEVs, located on the patient’s left side) were carefully preserved. After developing the avascular plane between the gastric and colonic mesenteries, subsequent dissection was performed along the duodenal fascia to access Toldt’s space. Continued anterior dissection relative to the right perirenal fat pad resulted in complete liberation of the hepatic flexure. In cases of SIT, the duodenal position may be misinterpreted intraoperatively. The ligamentum teres hepatis has been demonstrated to provide consistent anatomical guidance for spatial reorientation in such anomalous configurations. During pancreatic head dissection, the classic trifurcation of Henle’s trunk (gastrocolic trunk), comprising the right gastroepiploic vein, anterior superior pancreaticoduodenal vein, and right colic vein (RCV, draining from the patient’s left side), was identified (**Figure 1F**). Given the SIT, the initial division of the ASPDV prevented pancreatic head bleeding. Subsequent dissection of the RCV was performed with left-sided retrograde traction along the SMV while preserving the RGEV. This approach provided optimal exposure of the middle colic vascular pedicle origin.

Next, we initiated the caudal approach by sharply dividing the cecum’s peritoneal reflections, developing the avascular space posterior to the distal ileum’s mesenteric insertion (**Figures 1G, H**). This plane was developed cephalad and medially to connect with the duodenal space and the cephalad dissection plane. Following mesenteric root visualization, nodal clearance was executed parallel to the superior mesenteric venous trunk (SMV) from its tributaries to the confluence (**Figure 1I**). The ileocolic vascular pedicle was initially identified and divided. Subsequent dissection along the SMV included sequential transection of two critical arterial branches: the right colic artery (RCA, supplying the patient’s left side) (**Figures 1J–L**) and the right branch of the middle colic artery (supplying the patient’s left side), achieving complete laparoscopic colon mobilization. An 8-cm left rectus muscle incision was made to extract the specimen (**Figure 2E** shows the specimen). The ileal transection was performed at the pre-ileocecal segment (20cm from the cecal base), with proximal colonic division maintained at a 20-cm tumor-free margin. The specimen was sent for pathological examination. A side-to-distal ileocolonic anastomosis was fashioned with a circular stapling device, while the colonic remnant was secured using a linear stapler. The anastomosis and stump were reinforced with Vicryl sutures. Finally, the surgical field was irrigated and inspected for hemostasis. We inserted the drain in the right subphrenic region before completing the fascial closure. The procedure was completed successfully. The procedure was completed in 180 min with an estimated 120 mL blood loss.

Pathology confirmed a T3N0M0 well-differentiated adenocarcinoma (as presented in **Figure 2F**). The patient achieved uneventful recovery with disease-free status maintained throughout the 12-month follow-up period.

## Discussion and conclusion

SIT, an uncommon developmental anomaly, results from embryological inversion of visceral situs, resulting in an inverted anatomical arrangement (3, 4). Hongsen et al. demonstrated that female patients exhibit a significantly higher incidence of colorectal cancer with SIT, suggesting that female sex is an independent risk factor influencing the prevalence of SIT-associated colorectal malignancies (5). It is not considered a predisposing factor for malignant transformation, and its exact etiology remains unclear (6).

Estrogen receptor signaling is involved in gastrointestinal embryonic development and carcinogenesis. Estrogen receptor beta (ERβ), the predominant subtype in the colonic mucosa, has been demonstrated to exert tumor-suppressive effects by modulating epithelial cell proliferation and cell death (7). Although some studies suggest a potential female predominance in colorectal cancer with SIT, the proposition that loss of ERβ expression serves as the underlying mechanism remains speculative and arguably simplistic. It is unclear whether ERβ expression differs between colorectal tumors in SIT patients and those in the general population, as direct comparative data are lacking. Furthermore, the observed female predominance could be influenced by non-biological factors such as demographic biases or differences in healthcare-seeking behavior between sexes, rather than reflecting a true biological predisposition. Thus, while hormonally mediated mechanisms are plausible, this hypothesis requires validation through future studies specifically designed to compare ERβ expression and function in well-characterized SIT versus non-SIT tumor cohorts.

SIT is most commonly associated with primary ciliary dyskinesia (PCD), which is predominantly caused by mutations in genes affecting ciliary structure and function (e.g., DNAH5, DNAI1) (8, 9). Based on evidence that estrogen can influence ciliary function in other biological contexts, we hypothesize that abnormal estrogen levels during a critical developmental window may potentially disrupt the function of nodal cilia, thereby contributing to the pathogenesis of SIT in a manner that is not yet genetically defined.

The research team of Cui et al. postulates that the initial cardiac rotational movement may induce the spatial reorientation of abdominal organs during embryonic development (10). Experimental evidence implicates the Sonic hedgehog (SHH) morphogen in establishing organ laterality. Ectopic right-sided SHH signaling has been shown to cause dextrocardia through altered cardiac looping morphogenesis, ultimately resulting in situs inversus (11, 12). While the exact pathogenesis is undetermined, existing hypotheses primarily involve either genetic mutations during embryogenesis or familial genetic inheritance patterns. Current consensus maintains that SIT does not directly contribute to oncogenesis, although recent clinical observations indicate possible correlations with specific cancer types, potentially mediated by impaired KIF3 complex functionality (13). Dysfunction of the KIF3 complex impairs the normal transport of N-cadherin to the cell membrane, leading to aberrant accumulation of β-catenin in the cytoplasm. Excess β-catenin can translocate into the nucleus and activate the transcription of pro-proliferative genes, thereby potentially contributing to tumor initiation and progression (14).

Colorectal cancer with SIT increases surgical difficulty, particularly for laparoscopic procedures, which place higher technical demands on surgeons. The medical literature documents merely a single reported instance of cecal carcinoma coexisting with complete situs inversus. We reviewed 20 case reports, but only one case of cecal carcinoma with SIT has been documented, as shown in **Table 1**. Preoperative evaluation of anatomical variations is crucial for laparoscopic colectomy in SIT-associated colon cancer cases. Computed tomographic angiography combined with CT colonography provides essential anatomical delineation to facilitate surgical planning. Additionally, the optimal configuration of the surgical team’s positioning and trocar placement strategy is critical for procedural success.

**Table 1:** Case series analysis: colorectal neoplasms in the SIT cohort.

Researcher	Year	Tumor site	Hemorrhage volume (mL)	Procedure duration (min)	Treatment	Reference
Fujiwara et al.	2007	Ascending colon	60	191	Laparoscopic right colectomy	(15)
Jung Wook Huh et al.	2010	Rectum	120	250	Laparoscopic rectal resection	(16)
Hye et al.	2011	Hepatic flexure of the colon	NR	119	Laparoscopic right colectomy	(17)
Kim et al.	2011	Transverse and sigmoid colon	NR	NR	Open total colectomy with ileo-rectal anastomosis	(6)
Sumi et al.	2013	Transverse colon	230	402	Laparoscopic left colectomy	(18)
Marcelo Pb et al.	2014	Hepatic flexure of the colon	NR	NR	Open right hemicolectomy	(19)
Yaegas-hi et al.	2015	Sigmoid colon	13	189	Laparoscopic sigmoidectomy	(20)
Hirano et al.	2015	Cecal	NR	125	Laparoscopic right colectomy	(2)
Sasaki et al.	2017	Ascending colon	10	109	Laparoscopic right colectomy	(21)
Beibei Cui et al.	2019	Rectum	<50	210	Robotic-assisted rectal resection	(10)
Takeda et al.	2019	Sigmoid colon	NR	195	Laparoscopic sigmoidectomy	(22)
Kojima et al.	2019	Ascending colon	20	237	Laparoscopic right colectomy	(23)
Karabayo et al.	2019	Sigmoid colon	NR	NR	Laparoscopic sigmoidectomy	(24)
Chen et al.	2020	Sigmoid colon	NR	120	Laparoscopic sigmoidectomy	(25)
Q Xu et al.	2020	Transverse colon	NR	NR	Intestinal stent placement	(13)
Kasai et al.	2021	Rectum	Low	194	Robotic-assisted rectal resection	(26)
Kudo et al.	2021	Sigmoid colon	Low	243	Laparoscopic sigmoidectomy	(27)
Cheng et al.	2022	Transverse colon	33	115	Open left hemicolectomy	(28)
Zheng et al.	2022	Transverse colon	50	240	Laparoscopic left hemicolectomy	(29)
Ji-Long et al.	2022	Transverse colon	50	178	Laparoscopic left hemicolectomy	(30)
Sa et al.	2024	Rectum	40	NR	Laparoscopic rectal resection	(31)
Junki et al.	2024	Ascending colon	5	168	Robotic-assisted right hemicolectomy	(32)
Midori et al.	2025	Stomach and sigmoid colon	71	535	Robotic-assisted combined gastrectomy and sigmoid colectomy	(33)
Mamoru et al.	2025	Sigmoid colon and rectum	NR	221	Laparoscopic low anterior resection	(34)

CT of the abdomen not only confirms the features of situs inversus but also enables accurate staging of colorectal cancer, including the assessment of serosal invasion and detection of suspicious regional lymph node metastases. Furthermore, CT colonography clearly delineates the spatial relationship between the tumor and adjacent organs, while also identifying characteristic signs of intestinal malrotation, such as an abnormally positioned ligament of Treitz and abnormal fixation of the horizontal duodenum (28). Moreover, preoperative vascular assessment holds particular clinical significance in patients with SIT complicated by colorectal cancer. Computed tomography angiography (CTA) enables precise evaluation of key vascular variations, with mirror-image configuration of the mesenteric arteries being the most common. In patients with SIT and right-sided colon cancer, vascular anatomy often includes the following variations: the SMA being typically located on the patient’s left side (15, 21, 30), absence of the RCA (23, 25), and the ICA coursing ventral to the SMV (15, 23). Consequently, intraoperative adjustments are required, including modified trocar placement and mirror-image instrument setting. The surgeon’s position should be opposite to that in conventional procedures, and the camera port should be symmetrically repositioned. Additionally, appropriate adjustments in patient positioning and operating team layout are also necessary. When the RCA is absent and the ICA exhibits mirrored variation, the strategy adopted by the team of Kojima et al. involves prioritizing dissection at the mirror-image location of the SMA root followed by direct ligation of the vessel to ensure operative safety (23). In patients with SIT accompanied by left-sided colon cancer, sigmoid colon cancer, or rectal cancer, it is relatively common for the inferior mesenteric artery (IMA) to originate from the right side of the abdominal aorta (16, 20, 26). Kasai et al. recommend that intraoperative priority should be given to handling the IMA root: through retroperitoneal dissection, its origin is clearly identified on the right side of the abdominal aorta to avoid vascular injury caused by blind separation (26). This strategy can significantly enhance surgical safety. Performing CTA of the IMA and its branches can clearly demonstrate variations in vascular course and branching patterns, thereby providing crucial anatomical evidence for intraoperative decision-making regarding whether to preserve the left colic artery (LCA). Kudo et al., during a laparoscopic procedure on a patient with SIT and sigmoid colon cancer, observed that compared to non-SIT patients, right-handed surgeons often struggle to complete the separation of the splenic flexure from the pancreas and spleen via a cephalad approach due to mutual interference among laparoscopic instruments (27). However, in SIT patients, adopting a right-sided surgeon position combined with an auxiliary suprapubic port allows more efficient mobilization of the splenic flexure. Their study indicated that the use of an additional auxiliary trocar can significantly improve both the safety and convenience of the procedure. Besides adjusting positions, the surgeon’s operative habits also influence the fluency of the surgery. Enhanced operative ergonomics were reported for left-dominant surgeons during minimally invasive procedures in SIT patients, as documented by Oms and Badia’s research team (35).

In patients with SIT complicated by right-sided colon cancer, as the main operating field shifts to the patient’s left abdominal cavity, the conventional right-sided main operating port position causes instruments controlled by right-handed surgeons to form large angles when reaching left-sided target areas. This not only restricts freedom of movement but also easily leads to instrument interference. When standing on the patient’s right side, the instruments controlled by their dominant hand (left hand) can be naturally directed toward the patient’s left abdominal cavity. This posture significantly reduces arm crossing amplitude, decreases the likelihood of instrument collision, and enables smoother and more stable fine dissection within confined spaces.

Nevertheless, right-handed operators can achieve comparable outcomes through appropriate positional adjustments: 1) adjusting positioning: the surgeon moves between the patient’s legs or to the left side of the patient to improve the instrument axis toward the left operating area; 2) role swapping: a left-handed assistant takes on the main dissection tasks in the patient’s left quadrant; and 3) trocar layout optimization: an additional 5-mm auxiliary trocar is placed on the patient’s left abdominal wall specifically for retraction or exposure assistance to the right-handed surgeon.

The robotic surgical system, as an emerging minimally invasive option, demonstrates distinct advantages in sphincter-preserving surgery for low rectal cancer that requires delicate anatomical manipulation. Cui et al. further indicated that the robotic system, equipped with 7-degree-of-freedom wristed instruments and intelligent tremor filtration, significantly improves operational accuracy within the narrow pelvic cavity—a feature particularly critical in performing precise procedures such as total mesorectal excision (TME) (10). However, robotic surgery is more expensive than both laparoscopic and open approaches. As reported by Cheng et al., open sigmoidectomy remains a feasible alternative for patients with limited financial capacity who cannot afford minimally invasive techniques (28). Moreover, in cases where severe intestinal dilation occupies most of the abdominal cavity and is difficult to alleviate, studies such as that of Hj et al. suggest that conversion to open surgery may be safer due to significantly restricted laparoscopic working space (17). These specific circumstances emphasize that clinical decision-making should comprehensively consider individual patient conditions and surgical safety rather than blindly pursuing minimally invasive technologies.

The procedure necessitates a combined cephalocaudomedial approach to optimize surgical safety. Technical considerations include 1) tactical plane marking: gauze placement as an anatomical reference when approach transition is required; 2) embryological plane preservation: meticulous dissection along Toldt’s fascia; and 3) vascular anatomy focus: standard identification of the right colic artery and Henle’s trunk, given the low incidence (<15%) of ileocolic/middle colic artery variations.

While the combined cephalocaudomedial approach represents a standardized and well-established technique in laparoscopic right hemicolectomy for patients with normal anatomy, its application in SIT requires deliberate cognitive reorientation of the surgical field. Although the anatomical sequence is mirror-reversed, the fundamental principles of early vascular control and mesocolic plane dissection remain paramount for ensuring both procedural safety and oncological efficacy. Below, we discuss the key similarities and differences: The core principle remains identical: achieving early vascular control by dissecting along the central axis of the SMV.

In patients with normal anatomy, the surgical dissection plane is developed along the right side of the SMV from the patient’s perspective, and Henle’s trunk is typically located on the right side of the SMV. Conversely, the anatomical relationships are entirely reversed in patients with SIT.

Regarding intraoperative anatomical navigation, conventional procedures rely on standard landmarks such as the right perirenal fat pad. However, in SIT cases, given the reversed anatomy, constant anatomical structures—such as the ligament of Treitz and the umbilical fissure—serve as critical landmarks for spatial orientation and surgical guidance.

This approach is especially critical in SIT: the mirror-image anatomy increases the risk of misidentifying vessels (mistaking the RCV for a middle colic branch) or injuring the pancreaticoduodenal complex. The combined approach mitigates this risk by 1) providing early exposure of the SMV axis, which serves as a fixed navigational roadmap despite mirrored anatomy; 2) allowing sequential control of vessels from a medial perspective; and 3) reducing reliance on variable external landmarks.

The surgical outcomes of our case were compared against the benchmark case of cecal carcinoma in SIT reported by Hirano et al. (2) and a pooled analysis of other documented laparoscopic procedures for colorectal cancer in SIT patients. Our operative time of 180 min was longer than the 125 min documented by Hirano et al. This disparity may be attributed to factors such as the initial learning curve associated with operating on mirror-image anatomy and the need for meticulous dissection due to vascular anatomical variations. Regarding intraoperative blood loss, our estimated blood loss of 120 mL was slightly higher than those cases reported for minimally invasive right colectomies in SIT patients, but it remains within acceptable limits for laparoscopic colorectal surgery and is considerably lower than the blood loss reported in open procedures. Our findings further substantiate that laparoscopic surgery for colorectal cancer in SIT is both feasible and safe, with outcomes largely dependent on the specific tumor location and the surgeon’s familiarity with mirrored anatomy.

Analysis of the compiled case series (**Table 1**) reveals several notable trends. First, regarding tumor location, there is an apparent predominance of malignancies in the sigmoid colon and rectum compared to the proximal colon among patients with SIT. The reason for this predominant distribution remains unclear. One plausible hypothesis is that the abnormal fixation and rotation of the gut in SIT could alter colonic motility and stool transit time, potentially leading to prolonged mucosal exposure to carcinogens in the distal colon. However, this theory requires further investigation through comparative physiological studies. Second, concerning surgical outcomes, the data suggest that laparoscopic resection is a feasible and safe approach for patients with SIT, with conversion rates and complication profiles that appear comparable to those observed in patients with normal anatomy.

Although the reversed anatomy in SIT patients presents inherent technical challenges for laparoscopic surgery, this minimally invasive approach can still achieve comparable safety and efficacy to conventional procedures when performed by experienced surgeons with adequate anatomical understanding.

## Method

Anatomical nomenclature in SIT: All descriptions of anatomical sidedness (such as “right” or “left”) in this report are based on the patient’s own perspective. Consequently, due to situs inversus totalis, the anatomical structures are mirrored. This means that the “right colon” and associated vessels were located in the patient’s left abdomen. Specifically, the vessels critical to this right hemicolectomy—namely, the artery and vein that would be termed the “right colic vessels” in standard anatomy and the “right gastroepiploic vessels”—were situated on the patient’s left side.

## Data Availability

The original contributions presented in the study are included in the article/supplementary material. Further inquiries can be directed to the corresponding author.
